# Identification of Gut Microbiota Affecting Fiber Digestibility in Pigs

**DOI:** 10.3390/cimb44100312

**Published:** 2022-09-30

**Authors:** Qing Niu, Guang Pu, Lijuan Fan, Chen Gao, Tingxu Lan, Chenxi Liu, Taoran Du, Sung Woo Kim, Peipei Niu, Zongping Zhang, Pinghua Li, Ruihua Huang

**Affiliations:** 1 Institute of Swine Science, Nanjing Agricultural University, Nanjing 210095, China; 2 Huaian Academy of Nanjing Agricultural University, Huaian, 223005, China; 3 Institute of Animal Husbandry and Veterinary Science, Shanghai Academy of Agricultural Sciences, Shanghai 201403, China; 4 Department of Animal Science, North Carolina State University, Raleigh, NC 27695, USA

**Keywords:** gut microbiota, apparent NDF digestibility, apparent ADF digestibility, microbial function

## Abstract

Dietary fiber plays an important role in porcine gut health and welfare. Fiber is degraded by microbial fermentation in the intestine, and most gut microbiota related to fiber digestibility in pigs are worth pursuing. The aim of this study was to identify gut microbiota associated with the apparent total tract digestibility (ATTD) of neutral detergent fiber (NDF) and of acid detergent fiber (ADF) in pigs. Large phenotypic variations in the ATTD of NDF and of ADF were separately found among 274 Suhuai pigs. Microbial community structures were significantly different between high and low fiber digestibility groups. Fourteen genera separately dominated the communities found in the high ATTD (H-AD) of NDF and ADF samples and were in very low abundance in the low ATTD (L-AD) of NDF and ADF samples. In conclusion, *norank_f__Bacteroidales_S24-7_group* (*p* < 0.05), *Ruminococcaceae_UCG-005* (*p* < 0.05), *unclassified_f__Lachnospiraceae* (*p* < 0.05), *Treponema_2* (*p* < 0.01), and *Ruminococcaceae_NK4A214_group* (*p* < 0.01) were the main genera of gut microbiota affecting the ATTD of NDF in pigs. *Christensenellaceae_R-7_group* (*p* < 0.01), *Treponema_2* (*p* < 0.05), *Ruminococcaceae_NK4A214_group* (*p* < 0.05), *Ruminococcaceae_UCG-002* (*p* < 0.05), and *[Eubacterium]_coprostanoligenes_group* (*p* < 0.05) were the main genera of gut microbiota affecting the ATTD of ADF in pigs. The most important functions of the above different potential biomarkers were: carbohydrate transport and metabolism, general function prediction only, amino acid transport and metabolism, cell wall/membrane/envelope biogenesis, translation, transcription, replication, energy production and conversion, signal transduction mechanisms, and inorganic ion transport and metabolism. The most important metabolic pathways of the above different potential biomarkers were: membrane transport, carbohydrate metabolism, amino acid metabolism, replication and repair, translation, cell motility, energy metabolism, poorly characterized, nucleotide metabolism, metabolism of cofactors and vitamins, and cellular processes and signaling.

## 1. Introduction

Over the last 25 years, in order to improve performance, to save cost, and to reduce pig production pollutants, efforts have been made to formulate pig diets [1]. Fiber has the capacity to reduce ammonia emission and to improve gut health and pig welfare, and therefore, there has been research on fiber in the diet of pigs. In 1953, dietary fiber was identified as the non-digestible constituents of the plant cell wall; today, dietary fiber has been defined as non-digestible carbohydrates and lignin in plants [2]. Several fiber analysis procedures are available, which include the apparent digestibility of crude fiber (CF), neutral detergent fiber (NDF), and acid detergent fiber (ADF) methods. The ADF and NDF methods, as developed by Van Soest and Wine [3], have often been used for fiber analysis. Cellulose, hemicellulose, and lignin are the main components of NDF, and the major components of ADF include hemicellulose and lignin.

All animals are associated with a diverse microbial community that is mainly composed of bacteria [4]. Bacteria are essential for the breakdown of cellulose, since animal digestive enzymes cannot digest most complex carbohydrates and plant polysaccharides [5]. These carbohydrates and plant polysaccharides are metabolized by microbes to short-chain fatty acids (SCFAs), including acetate, propionate, and butyrate [6,7]. The ability of pigs to digest dietary fiber is mainly derived from their hindgut microbiota; 25% of pigs’ total energy is provided by bacterial fermentation end products in the colon [8,9,10,11]. The extent of absorption and utilization of the volatile fatty acids produced in the large intestines of pigs determine the acceptability of fiber [12]. However, most gut microbiota related to fiber digestibility is still unidentified. Our previous study described the dynamic distribution of pig gut microbiota and their relationship with apparent crude fiber digestibility in different growth stages [13]. It was shown that the genera *Collinsella* and *Sutterella* were correlated with the fermentation of NDF, and *Clostridium*, *Collinsella*, *Robinsoniella,* and *Turicibacter* were correlated with the fermentation of ADF. As the gut bacteria related to fiber digestibility are difficult to isolate and to culture in a traditional way, more microbiota associated with NDF and ADF digestibility need to be further verified and discovered with *16S rRNA* gene sequencing technology.

Modern commercial pigs show a relatively poor capacity to digest dietary fiber, while Chinese indigenous pigs have a stronger capacity to digest fiber [11,14,15]. In this study, the Suhuai pig, a synthetic Chinese breed that was derived from the Huai pig (Chinese indigenous pig, 25%) and the Large White (75%), was chosen as an experimental animal model [11].

Based on the above, it is hypothesized that there is high variation in fiber digestibility within a group of pigs, caused by carbohydrate transport and metabolism that is affected by differences in the gut microbiota structure. This study aimed to detect the phenotypic variation of fiber digestibility within pigs using the Suhuai breed, and further, to identify gut microbiota associated with the ATTD of NDF and ADF.

## 2. Materials and Methods

The study was conducted according to the guidelines of the Declaration of Helsinki, and approved by the Animal Care and Use Committee of the Nanjing Agricultural University. All procedures and the use of animals were carried out in accordance with Guidelines for the Care and Use of Laboratory Animals prepared by the Institutional Animal Welfare and Ethics Committee of Nanjing Agricultural University, Nanjing, China (certification no. SYXK (Su) 2017-0007).

### 2.1. Sample Collection in Animals

A total of 274 healthy Suhuai pigs were selected to collect fecal and diet samples (no disease or diarrhea happened one week before sampling) at 160 days old, from the Huaiyin Pig Breeding Farm, Huaian, China, under the same husbandry conditions. All pigs were selected according to a unified breed standard and fed with an antibiotic-free corn-soybean diet (Table S1). One month before sampling, antibiotics in the feed or for any therapeutic purposes were not provided for pigs.

Diet samples and approximately 200 g of each fecal sample were collected in plastic bags; fecal samples were mixing with 15 mL 10% sulfuric acid to be fixed on site. These samples were used for analyzing the ATTD. Each sample was individually collected in 2 mL centrifuge tubes without any treatment for *16S rRNA* gene sequencing. All samples were kept in an ice box for preservation and transportation, and then stored at –80 °C in the laboratory [16].

### 2.2. Chemical Analysis

Fecal samples from the Suhuai pigs were dried at 65 °C to a constant weight. The determination of NDF and ADF contents was performed using the ANKOM A200 filter bag technique (AOAC 962.09) [17]. Acid insoluble ash (AIA) was used as an indigestible marker to assess the digestibility of the dietary components (AOAC 942.05) [18]. The following equation was used to calculate the digestibility of each sample:

$${\mathrm{CAD}}_{D}\left(\%\right)=100\times \left(1-\frac{{\mathrm{DC}}_{F}\times {\mathrm{AIA}}_{D}}{{\mathrm{DC}}_{D}\times {\mathrm{AIA}}_{F}}\right)$$

where CADD represents the apparent dietary components digestibility, DCF represents the dietary component in feces, AIAD represents the AIA concentration in diet, DCD represents the dietary component in diet, AIAF represents the AIA concentration in feces.

### 2.3. 16S rRNA Sequencing and Bioinformatics Analysis

The gut microbiota population in the pigs with extremely high ATTD of NDF (*n* = 6) and of ADF (*n* = 6), and low ATTD of NDF (*n* = 6) and of ADF (*n* = 6) were analyzed. Samples from 21 Suhuai pigs were used for microbial diversity using *16S rRNA* gene sequencing, three of which with L-AD NDF and L-AD ADF. Fecal microbial DNA was isolated with a Soil DNA Kit (Omega, D5625-01). Afterwards, the DNA concentration was measured using a UV spectrophotometer (Eppendorf, Bio Photometer). The hypervariable V4 region of *16S rRNA* gene with the length of approximately 280 bp was targeted for sequencing [19]. The barcoded fusion forward primer was 520 F (5′-GCACCTAAYTGGGYDTAAAGNG-3′), and the reverse primer was 802 R (5′-TACNVGGGTATCTAATCC-3′). The PCR condition was as follows: initial denaturation at 98 °C for 5 min, 98 °C denaturation for 10 s, 50 °C annealing for 30 s, and 72 °C extension for 30 s, repeated for 25 cycles; final extension at 72 °C for 5 min. All samples were amplified in triplicate. The PCR product was extracted from 2% agarose gel and purified using an AxyPrep DNA Gel Extraction Kit (Axygen Biosciences, Union City, CA, USA) according to the manufacturer’s instructions, and quantified using a Quantus^TM^ Fluorometer (Promega, Madison, WI, USA).

The merged fastq files were exported to the Quantitative Insights into Microbial Ecology (QIIME) software [20]. Chimera identification and removal were performed using UCHIME [21] in mother [22]. The non-repeat sequences were extracted from the optimized sequences to reduce the redundant computation in the analysis of the intermediate process (http://drive5.com/usearch/manual/dereplication.html, accessed on 20 August 2022). Single sequences that did not repeat were removed (http://drive5.com/usearch/manual/singletons.html, accessed on 20 August 2022). Similar sequences were clustered into OTUs using the seed-based uclust algorithm 24 at a 97% identity threshold [22]. Taxonomic identification was assigned using an RDP classifier [23,24]. Taxonomy was assigned using the Silva (Release128 http://www.arb-silva.de, accessed on 29 September 2016). Venn diagrams and rank abundance distribution curve were performed by using Mothur.

### 2.4. Statistical Analysis

The apparent fiber digestibility was calculated using the SAS 9.4 software [25]. Alpha diversity was calculated using Mothur [22]. The Wilcoxon rank-sum test was used to evaluate group differences in bacterial composition [26]. Pair-wise phylogenetic distance was measure by weighted UniFrac [27] to compare community compositions across samples. Principal co-ordinates analysis (PCoA) were used compress dimensionality into 2D principal coordinate analysis plots [28], enabling visualization of sample relationships. PICRUSt was used to explore the functional composition that the bacterial community data might convey [29].

## 3. Results

### 3.1. Variation of Apparent NDF and ADF Digestibility within Pigs

Data regarding the ATTD NDF and ADF of the 274 Suhuai pigs are shown in Table 1. The mean ± the SE of the ATTD of NDF was 70.4 ± 0.6 and that of the ATTD of ADF was 62. 9 ± 1.2. The variable coefficient (CV) of the ATTD of NDF was 12.1% and that of the ATTD of ADF was 18.8%.

### 3.2. Comparison of Apparent NDF and ADF Digestibility between High- and Low-AD Pigs

The average ATTD of NDF of six H-AD pigs was 83.30%, 30.20% more than that of the six L-AD pigs (*p* < 0.01, Table 2). The average ATTD of ADF of six H-AD pigs was 75.69%, 33.76% more than that of the six L-AD pigs (*p* < 0.01, Table 2).

### 3.3. DNA Sequence Data and Bacterial Community Structure of the ATTD of NDF and of ADF Samples between H- and L-AD Pigs

More than one million sequences were obtained from all samples, and there were 38,973 high quality sequences per sample with a range from 29,641 to 49,819. The average sequence length was 240 bp. A total of 927 OTUs were identified from the H- and the L-AD of NDF, core OTUs comprised approximately 94% of the total OTUs (Figure S1A), while 45 and 11 OTUs were characteristically shown in the H- and the L-AD of NDF, respectively. At the same time, a total of 935 OTUs were identified from the H- and the L-AD of ADF, core OTUs comprised approximately 92% of the total OTUs (Figure S1B), while 49 and 26 OTUs were characteristically showed in H- and L-AD of ADF, respectively.

Shannon and Simpson indexes were significantly different between the H- and the L-AD of NDF samples (*p* < 0.05, Table S2).

**Figure 1:**
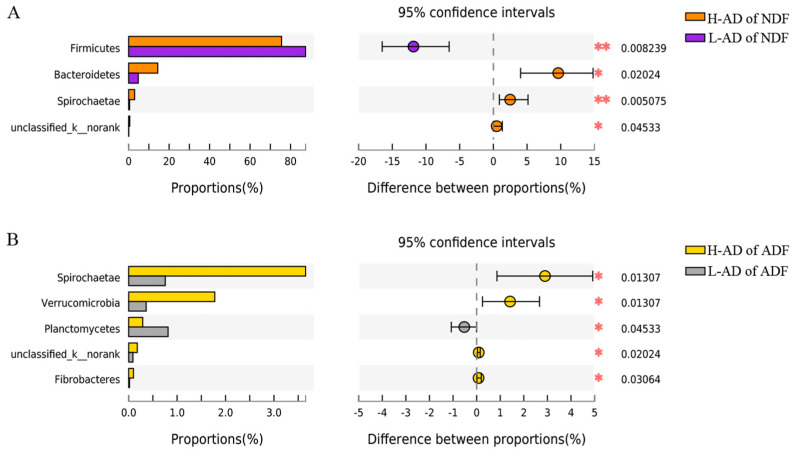
The bacterial abundances of distinct phyla significantly different between H- and L-AD of NDF (**A**) and ADF (**B**). Extended error bar plot showing phyla that had significant differences between H- and L-AD of NDF and ADF. Positive differences in mean relative abundance indicate phyla overrepresented on H-AD of NDF and ADF, while negative differences indicate greater abundance in L-AD of NDF and ADF. * The mean difference is significant at a level of 0.05, ** The mean difference is significant at a level of 0.01.

Fourteen phyla were identified from the four groups (Figure S2A,B): *Firmicutes*, *Bacteroidetes*, *Actinobacteria*, *Tenericutes*, *Spirochaetae*, *Verrucomicrobia*, *Proteobacteria*, *Planctomycetes*, *unclassified_k_norank*, *Saccharibacteria*, *Cyanobacteria*, *Chlamydiae*, *Fibrobacteres*, and *Lentisphaerae*. *Firmicutes* and *Bacteroidetes* were the most predominant phyla in all samples and comprised more than 91% of the total sequences. The abundances of *Bacteroidetes*, *Spirochaetae*, and *unclassified_k_norank* were significantly different between H-AD of NDF and L-AD of NDF (*p* < 0.05, Figure 1A). In contrast, the abundances of *Spirochaetae*, *Verrucomicrobia*, *unclassified_k__norank*, and *Fibrobactere* were significantly different between the H-AD of ADF and the L-AD of ADF (*p* < 0.05, Figure 1B).

At the genus level, 189 genera were identified from the NDF samples, and 182 of those existing were defined as core genera, while six and one genera were uniquely identified in the H- and the L-AD of NDF, respectively (Figure S3A). Meanwhile, 190 genera were distinguished from the ADF samples, and 183 of those existing were defined as core genera, while five and two genera were uniquely identified in the H- and the L-AD of ADF, respectively (Figure S3B). The two most dominant genera were *Lactobacillus* and *Streptococcus,* belonging to the phylum *Firmicutes*, which comprised more than 27.4% and 8.9% of the total sequences in the H- and the L-AD of NDF, respectively (Figure S2C). The two most predominant genera in the H- and the L-AD of ADF, separately containing about 26.7% and 8.4% of the total sequences, were *Lactobacillus* and *Christensenellaceae_R-7 group,* also belonging to the phylum *Firmicutes* (Figure S2D).

**Figure 2:**
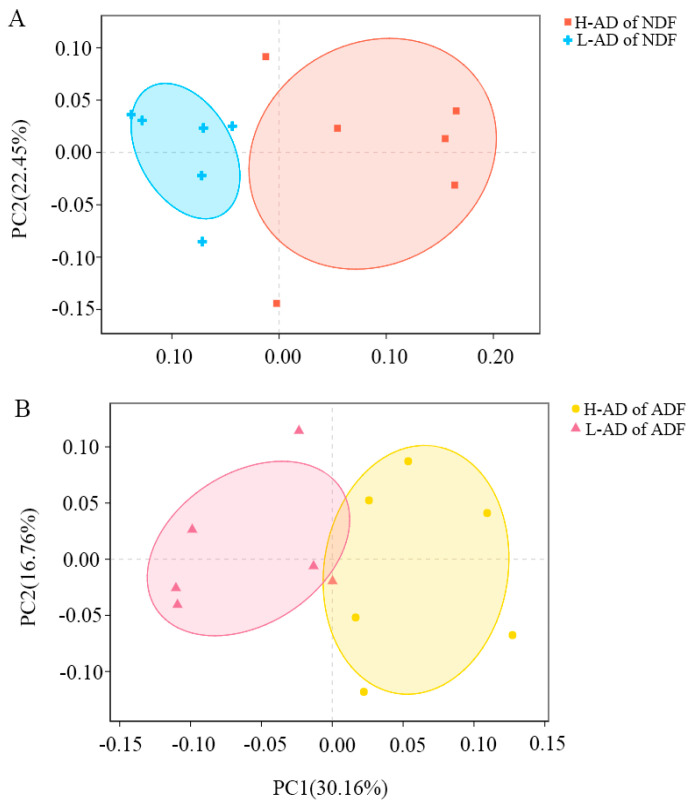
PCoA of H- and L-AD of NDF and ADF samples, respectively. PCoA was generated by using weighted UniFrac distance between H- and L-AD groups in NDF and ADF. The first principal coordinate separated H- and L-AD of NDF samples, explained 51.49% of sample variation (**A**). The first principal coordinate, explained 30.16% of sample variation, separated H- and L-AD of ADF samples (**B**).

The compositions of microbiota in the H-AD of NDF and of ADF were separately different to that observed in the L-AD of NDF and ADF (Figure 2A,B, Adonis/PERMANOVA, *p* < 0.01, Figure S4A,B).

**Figure 3:**
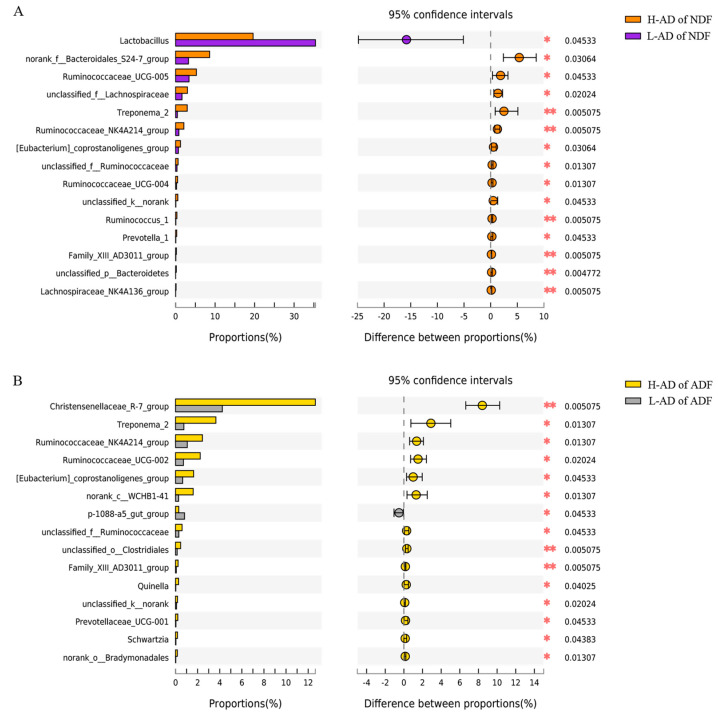
The bacterial abundances of distinct genera significantly different between the H- and the L-AD of NDF (**A**) and ADF (**B**). Extended error bar plot showing the fifteen most abundant genera that had significant differences between H- and L-AD of NDF and ADF. Positive differences in mean relative abundance indicate genera overrepresented on H-AD of NDF and ADF, while negative differences indicate greater abundance in L-AD of NDF and ADF. ** The mean difference is significant at a level of 0.01, * The mean difference is significant at a level of 0.05.

Relative abundance ranged as the top 15 differential genera were considered as potential biomarkers between the H- and L-AD groups of NDF and ADF. Fourteen genera were separately dominating the communities found in the H-AD of the NDF and ADF samples and were in very low abundance in the L-AD of the NDF and ADF samples (Figure 3A,B). There was a significant increase in the relative abundance of six genera (H-NDF∩H-ADF, *[Eubacterium]_coprostanoligenes_group*, *Family_XIII_AD3011_group*, *Ruminococcaceae_NK4A214_group,* and *unclassified_f__Ruminococcaceae* belong to the phylum *Firmicutes*; *Treponema_2* belongs to the phylum *Spirochaetae*; *unclassified_k__norank* belongs to the phylum *unclassified_k__norank*) in the H-AD samples as compared with the L-AD samples (Figure 3A,B). Samples had significant enrichment for eight genera (H-NDF, *Prevotella_1, norank_f__Bacteroidales_S24-7_group* and *unclassified_p_Bacteroidetes* belong to the phylum *Bacteroidetes*; *Lachnospiraceae_NK4A136_group*, *Ruminococcaceae_UCG-004*, *Ruminococcaceae_UCG-005*, *Ruminococcus_1* and *unclassified_f_Lachnospiraceae* belong to the phylum *Firmicutes*) only in the H-AD of the NDF group as compared with the L-AD of the NDF group (Figure 3A). Samples had significant enrichment for eight genera (H-ADF, *Prevotellaceae_UCG-001* belong to the phylum *Bacteroidetes*; *Christensenellaceae_R-7_group*, *Quinella*, *Ruminococcaceae_UCG-002*, *Schwartzia* and *unclassified_o_Clostridiales* belong to the phylum *Firmicutes*; *norank_o_Bradymonadales* belongs to the phylum *Proteobacteria*; *norank_c_WCHB1-41* belongs to the phylum *Verrucomicrobia*) only in the H-AD of the ADF group as compared with the L-AD of the ADF group (Figure 3B).

### 3.4. Prediction Functions of Microbial Metabolism

Twenty three functions were predicted in the present study. The most enriched functions were: general function prediction only (8.40%), carbohydrate transport and metabolism (8.37%), amino acid transport and metabolism (8,31%), replication (8.02%), translation (7.79%), transcription (7.59%), cell wall/membrane/envelope biogenesis (6.50%), energy production and conversion (5.50%), inorganic ion transport and metabolism (5.23%) and signal transduction mechanisms (4.95%). At the same time, 39 metabolic pathways were predicted and the following were the most enriched pathways: membrane transport (12.97%), carbohydrate metabolism (10.42%), replication and repair (9.69%), amino acid metabolism (9.16%), translation (6.47%), energy metabolism (5.54%), poorly characterized (4.81%), nucleotide metabolism (4.45%) and metabolism of cofactors and vitamins (4.04%).

According to the Clusters of Orthologous Groups of proteins (COG) and Kyoto Encyclopedia of Genes and Genomes (KEGG) databases, the top 10 in abundance predictive functions of potential biomarkers in the H-AD of NDF and ADF are shown in Table 3. The top 10 in abundance microbial metabolic pathways of potential biomarkers in the H-AD of NDF and ADF are shown in Table 4. The most important functions and metabolic pathways of the above different potential biomarkers included carbohydrate transport and metabolism and carbohydrate metabolism, respectively.

## 4. Discussion

Growing evidence suggests that a fiber-rich diet is one of the critical factors that contributes to the overall health and maintenance of a diverse healthy gut microbiota [30]. Therefore, it is important to identify gut microbiota related to the fiber digestibility of pigs.

### 4.1. Chinese Indigenous Pig Showed Better Fiber Tolerance Characteristics as Compared with Foreign Varieties

Here, the Suhuai pig, one of the synthetic Chinese pig breeds, was chosen to identify microbes associated with fiber degradation. The average H-AD of NDF and ADF were separately 83% and 76%, whereas those of the L-AD were separately only 53% and 42%, differences of 30% and 34%, respectively. This demonstrated a large phenotypic variation in the ATTD of NDF and of ADF among Suhuai pigs. Le Sciellour et al. investigated the relationships between microbiota and apparent digestibility coefficients with respect to age and diet [31]. The average NDF digestibility of three pig breeds (Duroc, Large White, and Pietrain pigs) was 63.1% during the fourth period (age of 21–23 weeks). It revealed that Chinese indigenous pig showed better fiber tolerance characteristics as compared with foreign pig varieties. To reveal gut microbiota associated with fiber digestibility, a comparative analysis of gut microbiota community structures was conducted on the H- and the L-AD of NDF and ADF, respectively.

### 4.2. Bacterial Community Structures Were Significantly Different between High and Low Fiber Digestibility Groups

A variety of commensal bacteria exist in animal large intestine, and they participate in many physiological processes beneficial to the host [32]. In the present study, the data showed a large microbial community in the Suhuai pigs. More than one million sequences were obtained from all samples, and there were 38,973 high-quality sequences per sample, with a range from 29,641 to 49,819. A total of 927 and 935 OTUs were identified from the NDF and ADF fecal samples, respectively. At the phylum level, *Firmicutes* and *Bacteroidetes* were the most predominant phyla, and comprised more than 90% of the total sequences, which was consistent with previous researches [13,33,34,35]. At the genus level, the two most predominant genera were *Lactobacillus* and *Streptococcus* which belong to the phylum Firmicutes in the NDF group. The two most predominant genera in the ADF group were *Lactobacillus* and *Christensenellaceae_R-7 group,* which also belong to the phylum *Firmicutes*. In our previous study, *Lactobacillus*, which comprised 15% of the total sequences, was the most dominant genera [13]. Regardless of the breed, *Prevotella*, *Blautia*, *Oscillibacter*, and *Clostridium* were generally prevalent in pig gastrointestinal tract [35]. In the research by Pu et al., *Firmicutes*, *Bacteroidetes,* and *Proteobacteria* were three dominant phyla in two intestinal locations (caecum and colon) at the phylum level; at the genus level, *Lactobacillus* and *Ruminococcaceae_UCG-005* were the top two genera in caecal samples, while Lactobacillus and Streptococcus were the top two genera in colonic samples [11]. Crespo-Piazuelo et al. described the microbiome composition, distribution, and interaction along the Iberian pig intestinal tract and its role in wholebody energy homeostasis. They showed that the *Prevotella* genus was the most dominant in the colon, representing 40.90% in the proximal part and 34.99% in the distal part [36]. The reasons that lead to different results with similar studies are complex. Many factors can cause different microbiome compositions (e.g., pig breeds, nutritional level, and sample selection).

The data between the H- and L-AD groups analyzed by Adonis/PERMANOVA showed statistical significance [37]. Microbial composition had a strong difference between the H- and the L-AD of NDF and ADF (adonis/PERMANOVA *p* < 0.01). The result illustrated that the gut microbiota between the H- and L-AD of the NDF and ADF samples were statistically significant and all data comparisons made between different groups in this study were meaningful. Although there were no differences in Chao and ace indexes between the H- and L-AD groups of this study, Shannon and Simpson indexes were significantly different between the H- and L-AD of NDF samples. The PCA of UniFrac distance matrices showed that the variation between H- and L-AD was primarily explained by the apparent NDF and ADF digestibility, respectively. This suggested that the differences of bacterial community structure between H- and L-AD were related to apparent NDF and ADF digestibility. However, these diversity indexes only showed the overall situation of microbiota in each group. As the objective of the present study was to reveal gut microbiota associated with apparent NDF and ADF digestibility, we needed to discover the microbiota with higher abundance in the H-AD and to predict their microbes functions.

### 4.3. Carbohydrate Transport and Metabolism and Carbohydrate Metabolism Pathway Were One of the Most Important Functions and Pathways of the Potential Biomarkers

Fiber is one of the main dietary components affecting the gut microbiota, and it consists of indigestible carbohydrates. High-fiber diets are associated with different positive metabolic effects and a diverse, healthy microbiota [38]. Fourteen genera separately dominated the communities found in the high ATTD (H-AD) of the NDF and ADF samples and were in very low abundance in the low ATTD (L-AD) of the NDF and ADF samples. Six of these genera were uniquely enriched in both H-AD of NDF and ADF as compared with L-AD of NDF and ADF, respectively. Eight genera were uniquely enriched only in H-AD of NDF and ADF, respectively. The H- and L-AD of the NDF and ADF samples showed different microbial community structure. In previous studies, scientists have found that high fiber diets could increase certain bacterial abundances. Simpson et al. showed that agrarian diets high in fruit/legume fiber were associated with greater microbial diversity and a predominance of *Prevotella* over Bacteroides [39]. In our current study, two numbers of *Prevotella* (*Prevotella_1* and *Prevotella_2*) were identified as potential biomarkers associated with apparent NDF and ADF digestibility. Makki et al. also came to a similar conclusion that the abundance of *Prevotella* was associated with long-term dietary intake of plant-based foods [40]. The results of Pu et al. revealed that the relative abundance of Ruminococcaceae_UCG-005 increased linearly with the increasing of dietary fiber level [11]. Some genera of *Ruminococcace* and *Lachnospiraceae* have been significantly, positively correlated with the intake of dietary fiber in the large intestine of pig. It has been reported that *Ruminococcaceae* and *Lachnospiraceae* could produce enzymes that degrade carbohydrates [41]. In our previous study, we showed that the bacterial abundance of *Treponema* was positively correlated with apparent crude fiber digestibility, which was consistent with the current study [13]. Since the large intestines of pigs have the strongest ability to degrade diet fiber with a mount of bacteria, other studies have also displayed gut bacteria associated with fiber digestibility, which was not significantly different in the current study. In Heinritz’s research [42], the abundances of *Lactobacilli*, *Bifidobacteria,* and *Faecalibacterium_prausnitzii* were higher and the abundance of *Enterobacteriaceae* was lower in a low-fat/high-fiber pig group. Tan et al. showed that the relative abundances of *Campylobacter* and *Butyricicoccus* were higher in cecum, and *Coprobacillus* was higher in colon [43]. Different bacteria degrade diets with different major fiber components. The genera of *Fibrobacter_intestinalis*, *Ruminococcus_flavefaciens*, *Ruminococcus_albus,* and *Butyrivibrio_spp.* are highly active cellulolytic bacterial species in pig gut, which are the dominant cellulolytic bacteria in the rumen [44]. *Prevotella_Bacteroides_ruminicola*, *F_sugginogenes*, *R_flavefaciens,* and *Butyrivibrio_spp.* are related to the hemicellulose fermentation process. Chen et al. [45] indicated that different fiber sources resulted in inconsistent microbiota composition in pig gut. Several genera, which were identified as potential biomarkers in the current study, have not been found related to dietary fiber digestibility. However, the potential biomarkers in the present study will provide a reference for further research on identification about gut bacteria associated with dietary fiber digestibility.

Dietary fiber is not hydrolyzed by human and animal digestive enzymes, but it is acted upon by gut microbes under anaerobic conditions. Metabolites such as short-chain fatty acids (SCFAs) are produced, such as acetate, propionate, and butyrate, which can be utilized by the host [46]. Fermentable dietary fiber, prebiotics, and probiotics contribute to increases in SCFAs via proliferation of beneficial SCFA-producing bacteria or fermentation of complex carbohydrates [47]. It has been estimated that, in pigs, 5–12% of the energy requirement is provided by bacterial fermentation end-products [8,10]. A previous study also found that SCFAs, especially butyrate, positively influenced host metabolism by activating intestinal gluconeogenesis, both in insulinsensitive and insulin-insensitive states, promoting glucose and energy homeostasis [48]. Marques et al. found that high consumption of fiber modified the gut microbiota populations and increased the abundance of acetate-producing bacteria [17]. Pu et al. also showed that as dietary fifiber increased, SCFAs production and microbial pyruvate metabolism and butanoate metabolism increased. Meanwhile, members of *Prevotellaceae*, *Ruminococcaceae,* and *Lachnospiraceae* have been linked to the fermentation of plant-derived non-starch polysaccharides to SCFAs [49]. As expected, a number of the predicted functions of these potential biomarkers in the H-AD of NDF and ADF were associated with microbial cell metabolism in the present study. Carbohydrate transport and metabolism was a very important microbial function of these potential biomarkers which were in high abundance in H-AD. In a previous study, they indicated that other abundant proteins from distal pig intestines have high sequence homology with the recognized carbohydrate membrane transport protein [50]. In their results, the most abundant SEED subsystem (MG-RAST annotation pipeline) was carbohydrate metabolism, which represented 13% of both pig fecal metagenomes. The above results show that gut microbes are closely related to utilization of various carbohydrates that play important roles in pig health.

## 5. Conclusions

A large phenotypic variation in the ATTD of NDF and ADF was observed within a group of Suhuai pigs. The microbial community structures were different between the high and low fiber digestibility groups. *Norank_f__Bacteroidales_S24-7_group* (LDA value = 4.62, *p* < 0.01), *Treponema_2* (LDA value = 4.16, *p* = 0.01), *Ruminococcaceae_UCG-005* (LDA value = 3.87, *p* = 0.02), *unclassified_f__Lachnospiraceae* (LDA value = 3.84, *p* = 0.01), and *Ruminococcaceae_NK4A214_group* (LDA value = 3.82, *p* = 0.01) were the main genera of gut microbiota affecting the ATTD of NDF in pigs. *Christensenellaceae_R_7_group* (LDA value = 4.45, *p* = 0.02), *Treponema_2* (LDA value = 4.14, *p* < 0.01), *Ruminococcaceae_UCG_002* (LDA value = 4.01, *p* = 0.04), *norank_c__WCHB1_41* (LDA value = 3.82, *p* = 0.02), and *Ruminococcaceae_NK4A214_group* (LDA value = 3.77, *p* < 0.01) were the main genera of gut microbiota affecting the ATTD of ADF in pigs. The most important functions and metabolic pathways of the above different potential biomarkers included carbohydrate transport and carbohydrate metabolism.

## Figures and Tables

**Table 1:** Data regarding the ATTD of NDF and ADF.

ATTD	N	Range, %	Mean ± SE	CV, %
NDF	274	44.6–88.3	70.4 ± 0.6	12.1
ADF	274	30.0–83.1	62. 9 ± 1.2	18.8

**Table 2:** Comparison of the ATTD of NDF and of ADF between H- and L-AD pigs.

Group	H-AD		L-AD	
	N	Mean ± SE, %	N	Mean ± SE, %
NDF	6	83.3 ± 5.2 ^A^	6	53.1 ± 5.1 ^B^
ADF	6	75.7 ± 4.8 ^A^	6	41.9 ± 8.5 ^B^

^AB^ The mean difference is significant at a level of 0.01.

**Table 3:** The proportion of each group in the top 10 function abundance (%).

Functions	H-AD of NDF∩H-AD of ADF	H-AD of NDF	H-AD of ADF
Carbohydrate transport and metabolism	8.68	8.55	7.20
General function prediction only	8.01	7.95	8.25
Transcription	7.58	6.30	7.55
Amino acid transport and metabolism	7.42	7.94	8.71
Translation, ribosomal structure, and biogenesis	7.22	7.70	6.96
Replication, recombination, and repair	7.11	7.23	7.20
Cell wall/membrane/envelope biogenesis	7.00	9.25	6.04
Signal transduction mechanisms	6.26	4.50	6.50
Energy production and conversion	5.60	6.23	6.22
Inorganic ion transport and metabolism	5.00	5.91	5.24

**Table 4:** The proportion of each group in the top 10 metabolic pathways abundance (%).

Pathways	H-AD of NDF∩H-AD of ADF	H-AD of NDF	H-AD of ADF
Membrane transport	13.67	8.41	12.64
Carbohydrate metabolism	10.00	10.44	9.44
Amino acid metabolism	9.08	10.64	9.64
Replication and repair	8.79	9.70	8.78
Translation	6.23	6.33	5.88
Cell motility	5.47	-	4.45
Energy metabolism	5.26	6.69	5.65
Poorly characterized	5.01	4.74	4.49
Nucleotide metabolism	3.85	4.49	3.89
Metabolism of cofactors and vitamins	3.46	4.69	4.39
Cellular processes and signaling	-	3.80	-

## Data Availability

The *16s rRNA* gene sequencing raw data have been submitted to the NCBI BioProject with accession number PRJNA579010.

## Supplementary Materials

The following supporting information can be downloaded at: https://www.mdpi.com/article/10.3390/cimb44100312/s1, Figure S1A,B: Venn diagrams of the OTUs between different groups, Figure S2A–D: Phyla and genera distribution. Phyla and genera distributions as a percentage of the total number in NDF (A and B) and ADF (C and D) groups, respectively, Figure S3A,B: Venn diagrams of the genera between high and low groups of NDF and ADF, respectively, Figure S4A,B: Adonis/PERMANOVA analysis. Distance box plot in NDF (A) and ADF (B) groups, respectively, Table S1: OTU richness and diversity indexes were compared between H- and L-AD of NDF and ADF samples, Table S2: Composition and nutrient level of experimental diet.
