# Supplementary material for: Identification of Gut Microbiota Affecting Fiber Digestibility in Pigs
Source: Curr Issues Mol Biol. 2022 Sep 30;44(10):4557–69. doi: 10.3390/cimb44100312 (PMC9600093; doi:10.3390/cimb44100312)
Supplement: Supplementary file 1 [file cimb-44-00312-s001.zip › cimb-1905843-supplementary.pdf]

Supplemental material

Figure S1A,B Venn diagrams of the OTUs between different groups.

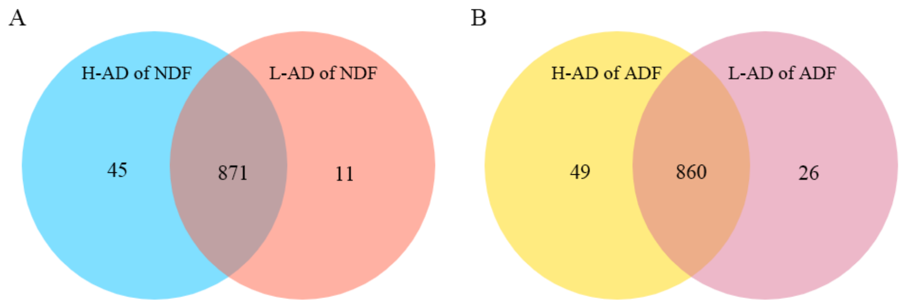

Figure S2A–D Phyla and genera distribution. Phyla and genera distributions as a percentage of the total number in NDF (A and C) and ADF (B and D) groups, respectively.

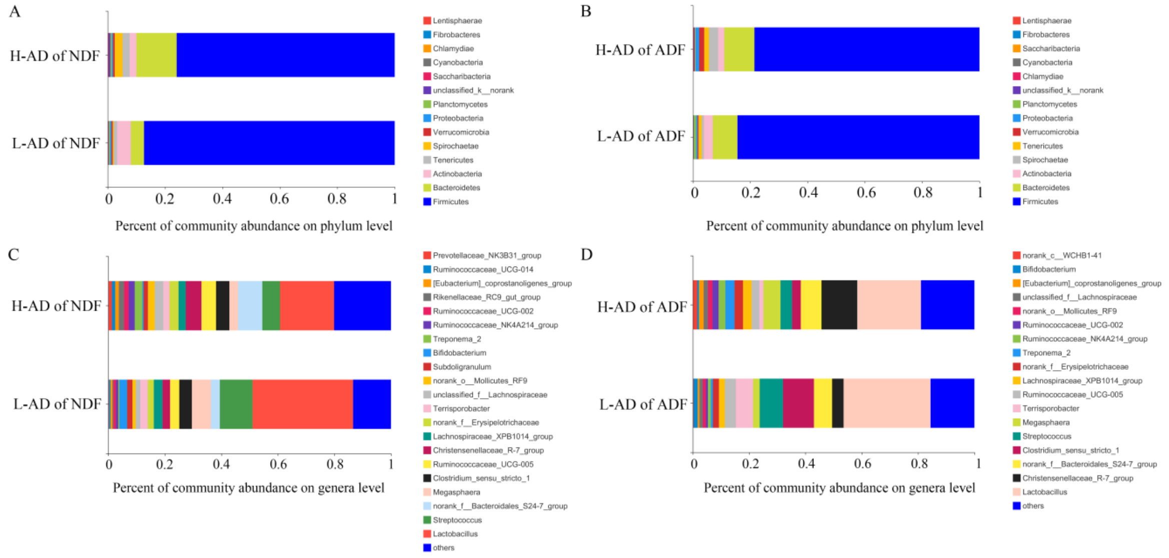

Figure S3A,B Venn diagrams of the genera between high and low groups of NDF and ADF, respectively.

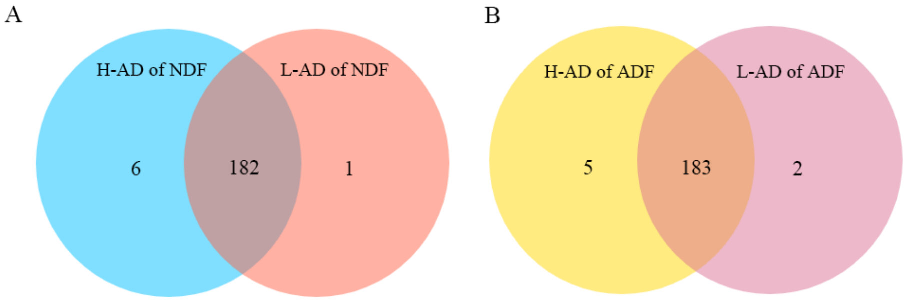

Figure S4A,B Adonis/PERMANOVA analysis. Distance box plot in NDF (A) and ADF (B) groups, respectively.

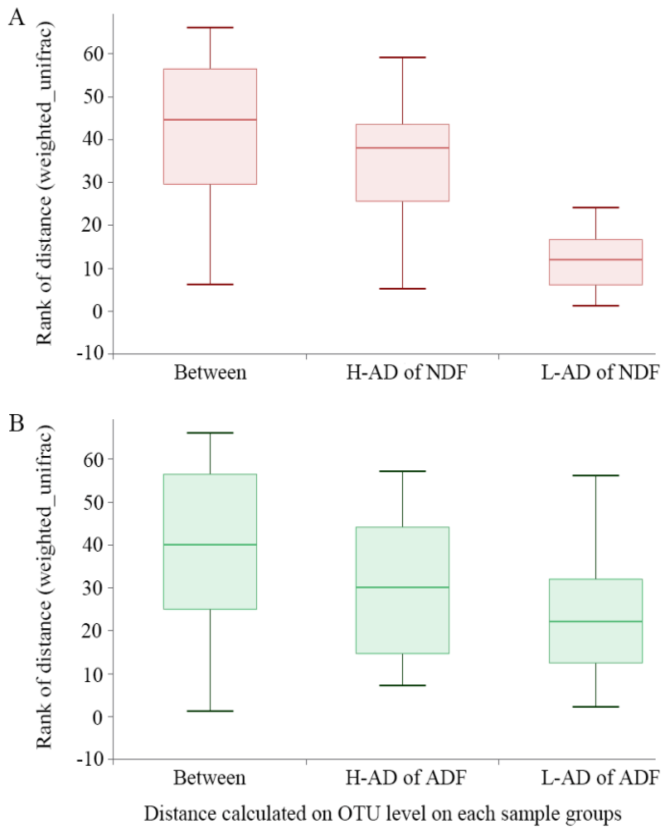

Table S1 Composition and nutrient level of experimental diet.

Diet composition	Content (%)
Corn	52.9
Wheat Bran	24
Soybean meal	15.9
Fish meal	2
Yeast powder	2
Premix	1
Salt	0.3
Stone powder	0.5
Lysine	0.1
Calcium hydrogen phosphate	0.12
Multivitamins	0.02
**Analyzed nutrient composition (%)**	
Crude protein	16.60
NDF	19.83
ADF	5.90
digestibleenergy(MJ/kg)	12.51

Premix: VA (KIU/kg): 500-700; VD3 (KIU/kg): 100-200; VE (IU/kg): ≥ 2000; VK3 (mg/kg): 75-800; VB1 (mg/kg): ≥ 75; VB2 (mg/kg): ≥ 400; VB6 (mg/kg): ≥ 100; VB12 (mg/kg): ≥ 2.5; Niacin (mg/kg): ≥3000; Pantothenic acid (mg/kg): ≥ 1000;

Folic acid (mg/kg): ≥ 50; Choline (g/kg): ≥ 10; Iron (g/kg): 5-10; Copper (g/kg): 0.6-1.2; Manganese(g/kg): 2.5-5.0; Zinc(g/kg):6-12; Iodine(mg/kg): 60-120; Selenium (mg/kg): 20-40; Water (%): ≤ 10.

Table S2 OTU richness and diversity indexes were compared between the two types in NDF and ADF groups.

	NDF		ADF	
	H-AD	L-AD	H-AD	L-AD
Shannon	4.74±0.21[A]	4.01±0.25[B]	4.46±0.26	4.05±0.30
Simpson	0.03±0.01[b]	0.06±0.02[a]	0.04±0.02	0.06±0.02
Ace	721.87±48.42	686.46±48.10	719.70±28.50	672.47±48.50
Chao	725.85±48.27	697.96±53.94	734.42±36.46	681.62±53.68

[AB] The mean difference is significant at a level of 0.01; [ab] the mean difference is significant at a level of 0.05.
